# Impact of the serum albumin level on acute kidney injury after cerebral artery aneurysm clipping

**DOI:** 10.1371/journal.pone.0206731

**Published:** 2018-11-05

**Authors:** Ji -Yeon Bang, Seon-Ok Kim, Sae-Gyeol Kim, Jun-Gol Song, Jiwon Kang, Jong-Wook Kim, Seungil Ha

**Affiliations:** 1 Department of Anesthesiology and Pain Medicine, Asan Medical Center, University of Ulsan College of Medicine, Seoul, Korea; 2 Department of Clinical Epidemiology & Biostatistics, Asan Medical Center, University of Ulsan College of Medicine, Seoul, Korea; University of Leicester Medical School, UNITED KINGDOM

## Abstract

**Background:**

Although hypoalbuminemia is a known risk factor for acute kidney injury (AKI) following surgery, little is known about its effects following aneurysm clipping surgery. We aimed to investigate the predictors of AKI and overall mortality and assessed the relationship between preoperative albumin and postoperative outcomes after aneurysm clipping surgery.

**Methods:**

This study included 2,339 patients who underwent aneurysm clipping surgery. According to the criteria updated by the Kidney Disease: Improving Global Outcomes (KDIGO), patients were classified into AKI and no AKI group. Independent AKI predictors were analyzed by multivariate methods, and the influence of AKI on the outcome variables was assessed with by propensity score matching analysis. Survival in relation to AKI was analyzed using the Kaplan–Meier method.

**Results:**

The total proportion of patients who developed AKI was 1.9%. The cutoff value of preoperative albumin for predicting AKI was 3.9 g/dL. Multivariate analyses showed that preoperative albumin≤ 3.9 g/dL, aneurysmal subarachnoid hemorrhage, male sex, phenylephrine use, and hemoglobin were associated with postoperative AKI development. In multivariate analysis, mortality was increased in AKI patients (p< 0.01). After propensity score matching, preoperative albumin≤ 3.9 g/dL was significantly related to AKI and overall mortality.

**Conclusion:**

Preoperative albumin≤ 3.9 g/dL is associated with postoperative AKI and mortality.

## Introduction

Perioperative acute kidney injury (AKI) is a common perioperative complication associated with increased morbidity and mortality [1, 2]. Recently, the Kidney Disease: Improving Global Outcomes (KDIGO) updated the diagnosing criteria for AKI [3]. Various studies have shown that AKI diagnosed based on the KDIGO criteria is associated with increased risk of morbidity and mortality [4, 5]. Because there is no curative treatment for AKI, it would be valuable to determine the modifiable risk factors for patients at increased risk of AKI [6].

One of the modifiable risk factors linked to increased risk of AKI is low albumin level [7, 8]. Albumin is not only responsible for plasma oncotic pressure, but is also a potential natural antioxidant that acts as a core extravascular source of reduced sulfhydryl groups [9]. These sulfhydryl groups, so-called thiols, scavenge reactive oxygen and nitrogen species [10], thereby causing postoperative AKI [11].

Although AKI has been reported as an independent predictor of outcomes after aneurysmal subarachnoid hemorrhage [12, 13], the influence of serum albumin on postoperative AKI development following cerebral artery aneurysm clipping has not been studied. We thus investigated whether preoperative albumin level is associated with postoperative AKI in patients after cerebral artery aneurysm clipping surgery.

## Methods

We retrospectively reviewed the electronic medical records and laboratory findings of 2,462 patients who underwent aneurysm-clipping surgery at Asan Medical Center between January 2008 and December 2014. Of these patients, 123 cases were excluded due to chronic kidney disease (n = 34) or incomplete laboratory results (n = 89). Thus, 2,339 patients were registered in the final study population. Among them, 357 patients had accompanying subarachnoidal hemorrhage (SAH). The institutional review board of Asan Medical Center waived the need for informed consent for this study due to its retrospective design and approved the study protocol (2015–0645).

### Clinical data

We retrospectively reviewed the computerized medical records at our center (Asan Medical Center Information System Electronic Medical Records) to obtain the demographic, laboratory, and intraoperative data on all patients and their postoperative outcomes. Demographic data included patient sex, age, comorbidities (hypertension, diabetes mellitus, and ischemic heart disease), body mass index, smoking history, and medications (beta-blocker, aspirin, calcium channel blocker, angiotensin-converting enzyme inhibitor, antiplatelet agent, and 3-hydroxy-3-methylglutaryl-coenzyme A reductase inhibitor). Aneurysmal SAH was assessed through a review of medical records. Hypertension and diabetes mellitus were described as use of any antihypertensive or hypoglycemic agents at admission, and ischemic heart disease as positive coronary angiography or compatible electrocardiographic or perfusion scan findings.

Laboratory data included preoperative hemoglobin, platelets, uric acid, albumin, sodium, potassium, serum creatinine (sCr), chloride, and the estimated glomerular filtration rate (eGFR). eGFR was measured from the preoperative sCr concentration based on the Modification of Diet in Renal Disease study equation for adult patients and adjusted for each 1.73 m^2^ of body surface area [14]. Intraoperative anesthetic data included the mean blood pressure, amount of administered crystalloid solution and mannitol, proportion of patients transfused (ex; red blood cells), total urine output during the operation, anesthetic time, and proportion of patients requiring furosemide and phenylephrine administration.

### Anesthetic management

Radial artery was cannulated for invasive arterial pressure monitoring with the administration of fentanyl 50–100 μg. Anesthesia was induced with a bolus intravenous administration of propofol 1.5–2.5 mg/kg. Propofol and remifentanil were then infused with effect site target-controlled infusion (TCI) using a commercially available 2-channel TCI pump (Orchestra, Fresenius Vial, Brezins, France). Endotracheal intubation was facilitated by a single bolus of rocuronium 0.8–1.0 mg/kg. During the maintenance of anesthesia, the target concentrations of propofol and remifentanil were 2.5 μg/mL and 7–9 ng/mL, respectively, maintaining a bispectral index (BIS VISTA, Aspect Medical System, Norwood, MA, USA) of 40–60. During anesthesia, 0.9% normal saline was administered. Until aneurysm clipping, fluid administration was restricted to the sum of 2mL/kg/h and hourly urine output. After aneurysm clipping, the infusion rate of normal saline was increased to 4 mL/kg/h and hourly urine output. If hypotension occurred without significant bleeding, phenylephrine was continuously infused to retain a mean blood pressure > 65 mmHg. If plasma hemoglobin level was reduced to less than 8 g/dL due to bleeding, packed red blood cells were transfused.

### Definition of outcomes

Outcome variables included postoperative AKI, hospital and intensive care unit (ICU) stay, and overall mortality. Postoperative AKI was identified on the basis of the KDIGO classification using changes in the sCr on postoperative days 1–7 compared with the baseline sCr, which was the most recent concentration measured prior to operation. In-hospital mortality was determined by reviewing the electrical medical records. To validate the complete follow-up data regarding mortality, information on the date of death was obtained from the National Population Registry of the Korea National Statistical Office by using the unique personal identification number of each patient.

### Statistical analysis

Continuous variables are expressed as mean ±standard deviation (SD) or median with interquartile range. All continuous variables were evaluated for normality using the Shapiro-Wilk test; the t-test or Mann-Whitney rank sum test were applied to inspect intergroup differences when appropriate. Categorical variables are expressed as numbers and percentages and were evaluated using the chi-square test or Fisher’s exact test. A receiver operating characteristic (ROC)curve analysis was conducted to determine the cut-off value for anticipating postoperative development of AKI.

Multivariate regression analyses were performed to determine the predictors of AKI. Multivariate analysis was conducted for each variable with *p*<0.1in the univariate analysis. In addition, a nested case control study comprising 679 patients with hypoalbuminemia (≤3.9 g/dL) and679 matched patients (>3.9g/dL) using propensity score (PS) was conducted to elucidate the influence of hypoalbuminemia on postoperative AKI. PS was calculated for each patient using age; sex; body mass index; diabetes mellitus; hypertension; ischemic heart disease; smoking history; aneurysmal SAH; calcium channel blocker, angiotensin-converting enzyme inhibitor, beta-blocker, aspirin, antiplatelet agent, and statin use; hemoglobin; platelet count; uric acid, sodium, potassium, creatinine, and chloride level; and eGFR. We subsequently used the derived PS to match 679 patients with a serum albumin ≤ 3.9 g/dL with patients with a serum albumin > 3.9g/dL at a ratio of 1:1 using greedy matching algorithms. Patients without corresponding matches were excluded. After all PS matches were performed, we assessed the balance in baseline covariates using standardized mean difference, t tests, and McNemar tests for continuous and categorical variables as appropriate. We analyzed all available data without the imputation of missing values. PS matching was performed with the SAS software package (version 9.1; SAS Institute Inc., Cary, NC, USA).

To assess the adjusted odds ratio (OR) and hazard ratios (HR) of the relationship between a low preoperative albumin level and outcome variables, a weighted logistic regression and multivariate Cox proportional hazard regression analysis was used. The adjusted variables are listed in Table 1. In the PS matching analysis, the association of a lower preoperative albumin level with mortality was evaluated with a weighted logistic regression with generalized estimating equations or Cox proportional hazards regression models with robust standard errors. The PS matching analysis was adjusted with mannitol, crystalloid, and vasopressor use, which were significantly different between the 2 groups. The discrimination of the model was assessed using C statistics (C = 0.750), while calibration was assessed using Hosmer-Lemeshow statistics (χ^2^ = 9.1944; df = 8; *p* = .33).

**Table 1:** Demographic, preoperative, and intraoperative characteristics of the study patients.

	Preoperative albumin level		P value	Preoperative albumin level (Propensity score matched patients)		P value	Standardized difference
	≤3.9 g/dL (n = 821)	>3.9 g/dL (n = 1,518)		≤3.9 g/dL (n = 679)	>3.9 g/dL (n = 679)		
Demographic variable							
Age, years	58.1 ± 10.4	55.3 ± 9.6	< .01	57.2 ± 10.2	57.2 ± 9.5	.88	.007
Sex, male	205 (30.0)	524 (34.5)	< .01	179 (26.4)	186 (27.4)	.66	.024
Body mass index, kg/m^2^	24.4 ± 3.4	24.5±3.2	.52	24.5 ± 3.4	24.6 ± 3.2	.42	.042
Diabetes	71 (8.7)	142 (9.4)	.57	64 (9.43)	75 (11.1)	.33	.058
Hypertension	336 (40.9)	709 (46.7)	< .01	283 (41.7)	287 (42.3)	.82	.012
Ischemic heart disease	46 (5.6)	52 (3.4)	.01	31 (4.6)	35 (5.2)	.62	.026
Smoking history	216 (26.4)	470 (31.2)	.02	180 (26.5)	197 (29.0)	.30	.057
aSAH	171 (20.8)	186 (12.3)	< .01	120 (73.6)	108 (15.9)	.37	.044
CCB	352 (42.9)	591 (38.9)	.07	276 (40.7)	274 (40.4)	.91	.006
ACEI	183 (22.3)	352 (23.2)	.62	153 (22.5)	155 (22.8)	.89	.007
Beta-blocker	134 (16.3)	172 (11.3)	< .01	100 (14.7)	94 (13.8)	.63	.024
Aspirin	79 (9.6)	155 (10.2)	.65	61 (9.0)	70 (10.3)	.41	.045
Antiplatelet agent	105 (12.8)	169 (11.1)	.24	79 (11.6)	84 (12.4)	.68	.022
Statin	133 (16.2)	315 (20.8)	< .01	118 (17.4)	128 (18.9)	.48	.04
Laboratory data							
Hemoglobin, g/dL	12.8 ± 1.4	13.6 ±1.4	<.01	13.1 ± 1.3	13.1 ± 1.3	.35	.041
Platelets, ×10^3^/μL	238.4 ± 63.5	243.3 ± 54.4	.05	240.7 ± 63.7	239.7 ± 55.4	.76	.016
Uric acid, mg/dL	4.5±1.4	4.7± 1.4	<.01	4.6 ± 1.4	4.6 ± 1.4	.91	.006
Albumin, g/dL	3.7 ± 0.3	4.3±0.2	<.01	-	-	-	-
Sodium, mmol/L	140.4 ± 2.9	140.7 ± 2.5	<.01	140.5 ± 2.9	140.6 ± 2.6	0.8	.013
Potassium, mmol/L	4.0 ± 0.4	4.1 ± 0.3	<.01	4.1 ± 0.4	4.1 ± 0.4	.63	.025
Creatinine, mg/dL	0.7 ± 0.2	0.8 **±** 0.2	<.01	0.7 ± 0.2	0.7 ± 0.2	.70	.018
Chloride, mmol/L	105.1 ± 3.0	103.7 ± 2.6	<.01	104.6 ± 2.6	104.6 ± 2.5	.87	.007
GFR, mL/min/1.73m^2^	74.6 ± 14.1	72.8 ± 12.8	<.01	74.2 ± 13.7	74.6 ± 13.6	.56	.031
Intraoperative data							
Mean blood pressure	63.6 ± 5.6	63.6 ± 5.7	.85	63.6 ± 5.5	63.5 ± 6.0	.68	.023
Crystalloid solution, L	1.9 (1.4–2.2)	1.9 (1.4–2.2)	.45	1.9 ± 1.1	2.0 ± 1.0	.03	.073
Mannitol, mL	73.6 (0–100)	86.0 (50–100)	<.01	66.9 ± 76.6	87.6 ± 72.0	<.01	.268
PRBC transfusion, n (%)	88 (10.7)	84 (5.5)	<.01	54 (8.0)	54 (8.0)	1.0	.000
FFP transfusion, n (%)	3 (0.4)	5 (0.3)	.99	2 (0.3)	5 (0.7)	.26	.073
Platelet transfusion, n (%)	5 (0.6)	5 (0.3)	.34	4 (0.6)	2 (0.29)	.41	.038
Urine output, L	0.6 (0.4–1.0)	0.7 (0.4–1.1)	.06	0.8 ± 0.5	0.8 ± 0.6	.24	.094
Anesthetic time, min	286.6 ± 80.8	285.5 ± 76.6	.99	283.2 ± 78.5	287.0 ± 75.5	.35	.047
Vasopressor use	343 (42)	406 (27)	<.01	315 (46.4)	183 (27.0)	<.01	.412
Furosemide, n (%)	14 (1.7)	20 (1.3)	.45	9 (1.3)	10 (1.5)	.82	.011

Values are expressed as mean ± SD, median (interquartile range), or n (%). aSAH, aneurysmal subarachnoid hemorrhage; CCB, calcium channel blocker; ACEI, angiotensin converting enzyme inhibitor; GFR, glomerular filtration rate; PRBC, packed red blood cells; FFP, fresh-frozen plasma

The cumulative survival rate between groups was analyzed using the Kaplan–Meier method, and alterations between curves were assessed using the log-rank test. All p values < 0.05 were considered statistically significant. Data manipulation and statistical analyses were performed using SAS or R software (version 2.10.1).

## Results

The median follow-up duration for the entire patient population was 3.8 years (interquartile range:2.1–5.4years). According to the ROC curve analysis, a cut-off value of preoperative albumin of 3.9 g/dL predicted the development of postoperative AKI (sensitivity 65.5%, specificity 65.9%). Of the 2,339 study patients, 821patients (35.1%) showed preoperative albumin level of ≤3.9 g/dL (3.7±0.3) and 1,518 patients had preoperative albumin level of >3.9 g/dL(4.3±0.2). Patients with lower preoperative albumin levels (≤3.9 g/dL) were older, more likely to be female, and showed higher incidence rates of IHD and aneurysmal SAH. In addition, patients with preoperative albuminlevels≤3.9 g/dL had lower levels of hemoglobin (12.8 ± 1.4 vs 13.6 ± 1.4), uric acid (4.5 ± 1.4 vs 4.7 ± 1.4), and sodium (140.4 ± 2.9 vs 140.7 ± 2.5), and higher level of chloride (105.1 ± 3.0 vs 103.7 ± 2.6)compared with patients with preoperative albumin levels>3.9 g/dL. Baseline eGFR was higher in patients with preoperative albumin levels of ≤3.9 g/dL as well. Intraoperative data showed that patients with preoperative albumin levels of ≤3.9 g/dL received lower amounts of mannitol and more frequently required transfusions and vasopressor infusion. The demographic and intraoperative data of the patients are presented in Table 1.

**Fig 1:**
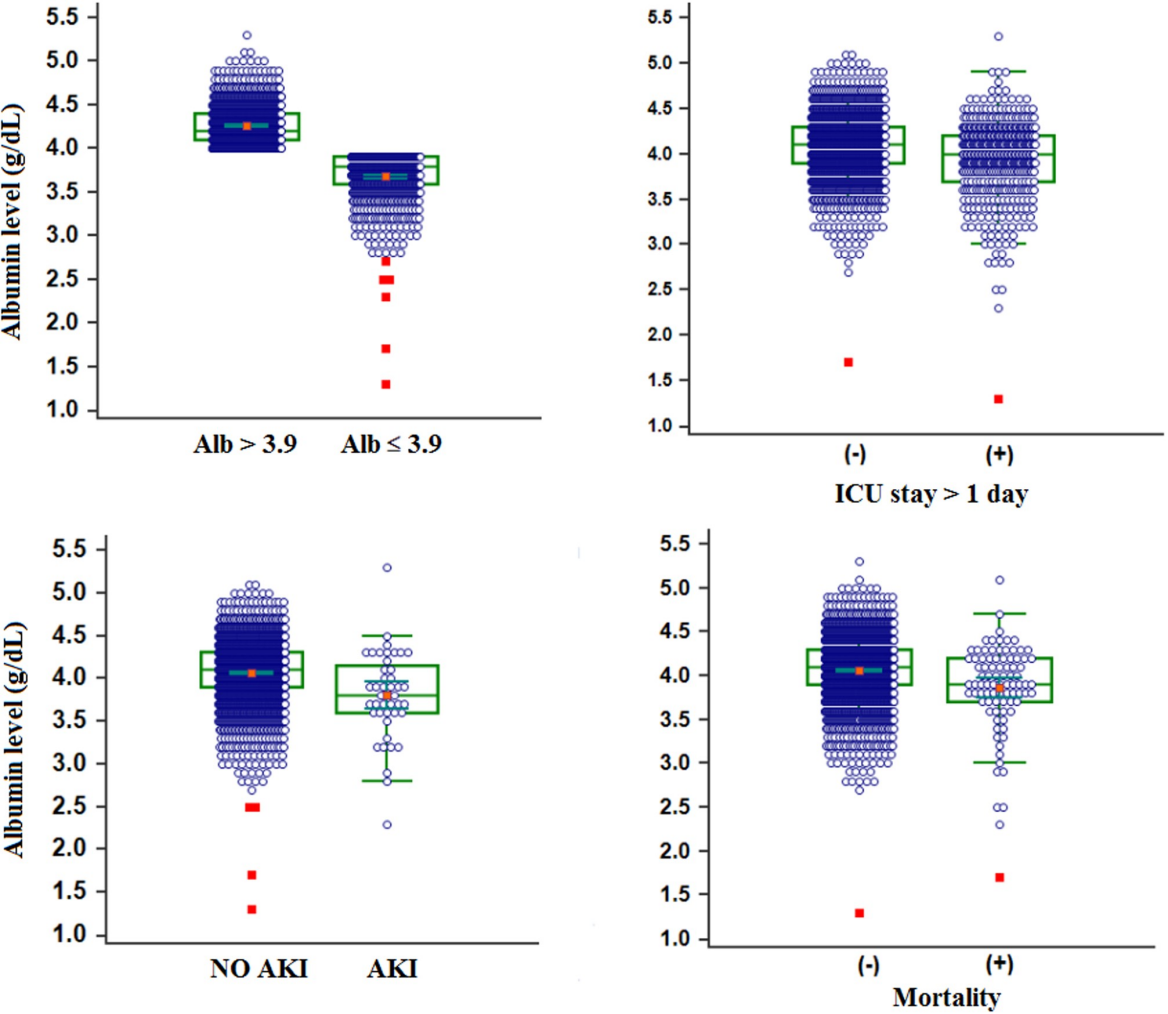
Preoperative albumin level according to the outcome variable. Central box represents the values from the lower to the upper quartile (25^th^ to 75^th^ percentile). The middle line represents the median. A line extends from the minimum to maximum value, excluding outliers. Patients with poorer outcomes such as AKI, ICU stay more than 1 day, and mortality had lower levels of albumin (p < 0.05 for all outcome variables). *; p < 0.05.

The overall proportion of patient who developed AKI was 1.9%. The rate of AKI development was higher in patients with lower preoperative albumin levels (3.5%; n = 29) compared with patients with higher preoperative albumin levels (1.0%; n = 15). Patients with lower preoperative albumin levels stayed longer in the hospital [7 (6–11) vs 6 (5–8) days; *p*< 0.001) and in the ICU (*p*< 0.001). Mortality was also higher in patients with lower preoperative albumin levels [47 (5.7%) vs 40 (2.6%); *p*< 0.001]. The preoperative albumin level according to the outcome variables is depicted in Fig 1.

Multivariate logistic regression revealed that the predictors of AKI were preoperative albumin level of ≤3.9 g/dL (OR 2.59, CI 1.31–5.13; p < 0.01), aneurysmal SAH (OR 3.91, CI 2.06–7.42; p <0.01), male sex (OR 3.39, CI 1.65–6.97; p<0.01), vasopressor use (OR 0.197, CI 0.057–0.676; p<0.01), and hemoglobin level (OR 0.72, CI 0.59–0.89; p< .01) (Table 2).

**Table 2:** Univariate and multivariate analysis of AKI predictors using the KDIGO criteria.

	Univariate				Multivariate			
	OR	95% CI		P value	OR	95% CI		P value
Sex (male)	1.86	1.023	3.397	.04	3.729	1.797	7.740	<.01
Hemoglobin, g/dL	0.728	0.596	0.888	<.01	0.691	0.563	0.849	<.01
Creatinine, mg/dL	0.021	0.003	0.163	<.01				
Preoperative albumin≤3.9g/dL	3.669	1.955	6.884	<.01	2.978	1.512	5.866	<.01
Mannitol, ml	1.007	1.004	1.011	<.01				
Crystalloid, L	1.000	1.000	1.000	<.01				
PRBC transfusion	3.871	1.879	7.976	<.01				
Aneurysmal SAH	5.851	3.204	10.684	<.01	3.570	1.898	6.712	<.01
Urine output, ml	1.001	1.000	1.001	<.01				
Vasopressor use	0.15	0.04	0.42	<.01	0.197	0.057	0.676	<.01
Anesthetic time,min	1.003	1.000	1.006	.03				

PRBC = packed red blood cells; SAH = subarachnoid hemorrhage

The predictive variables of overall mortality were age (HR1.08, CI 1.05–1.11; p<0.01), medication history of aspirin (HR0.29, CI 0.09–0.93; p = 0.04), aSAH (HR2.48, CI 1.56–3.93; p<0.01), red blood cell transfusion (HR2.00, CI 1.18–3.34; p = 0.01), urine output (HR1.0, CI 1.000–1.001; p<0.01), and AKI (HR2.38, CI 1.10–5.13; p = 0.03) (Table 3). In addition, we performed an analysis between the patients with SAH and without SAH. We summarized these results as a S1 Table.

**Table 3:** Univariate and multivariate analysis of overall mortality predictors.

	Univariate				Multivariate			
	HR	95% CI		P value	HR	95% CI		P value
Preoperative albumin ≤ 3.9 g/dL	2.429	1.592	3.706	<.01				
Age, yr	1.088	1.062	1.115	<.01	1.079	1.053	1.105	<.01
Mannitol, ml	1.003	1.000	1.006	.02				
CCB	1.578	1.036	2.403	.03				
Aspirin	0.314	0.099	0.992	<.05	0.290	0.090	0.932	.03
Aneurysmal SAH	3.423	2.228	5.260	<.01	2.480	1.564	3.933	<.01
Hemoglobin, g/dL	0.769	0.668	0.885	<.01				
Crystalloid, L	1.000	1.000	1.000	<.01				
MBP, mmHg	0.967	0.931	1.005	.09				
PRBC transfusion	4.647	2.917	7.403	<.01	2.003	1.184	3.387	.01
Urine output, ml	1.000	1.000	1.001	.02	1.000	1.000	1.001	<.01
AKI	5.357	2.684	10.692	<.01	2.376	1.102	5.126	.03

CCB = Calcium channel blocker; SAH = subarachnoid hemorrhage; MBP = mean blood pressure; PRBC = packed red blood cells.

The associations between preoperative albumin level ≤3.9 g/dL and clinical outcomes are shown in Table 4. Multivariate analysis revealed that preoperative albumin level ≤3.9 g/dL was related to AKI (OR2.59, CI 1.31–5.13; p <0.01), ICU stay (OR 1.82, CI 1.47–2.25; p<0.01), and hospital stay (OR 1.87, CI 1.56–2.23; p<0.01), but not to overall mortality (HR 1.43, CI 0.91–2.25; p = 0.12). After PS matching, preoperative albumin level ≤ 3.9 g/dL was independently associated with AKI (OR 2.80, CI 1.29–6.06; p < 0.01) and overall mortality (HR 1.90, CI 1.02–3.30, p = 0.04).

**Table 4:** Outcomes adjusted by preoperative albumin level ≤ 3.9 g/dL.

	Crude				Multivariate				PS matching^1^			
	**OR**	95% CI		P value	OR	95% CI		P value	OR	95% CI		P value
AKI	3.7	2.0	6.9	<.01	2.6	1.3	5.1	<.01	2.8	1.3	6.1	<.01
ICU stay > 1day	1.8	1.4	2.2	<.01	1.8	1.5	2.3	<.01	1.1	0.9	1.5	.3
Hospital stay >7 days	1.8	1.5	2.2	<.01	1.9	1.6	2.2	<.01	1.2	1.0	1.5	.1
	HR	95% CI		P value	HR	95% CI		P value	HR	95% CI		P value
Mortality	2.4	1.6	3.7	<.01	1.4	0.9	2.3	<.12	1.9	1.02	3.3	.04

^1^Adjusted by mannitol, crystalloid, and vasopressor use.

AKI = acute kidney injury; ICU = intensive care unit

**Fig 2:**
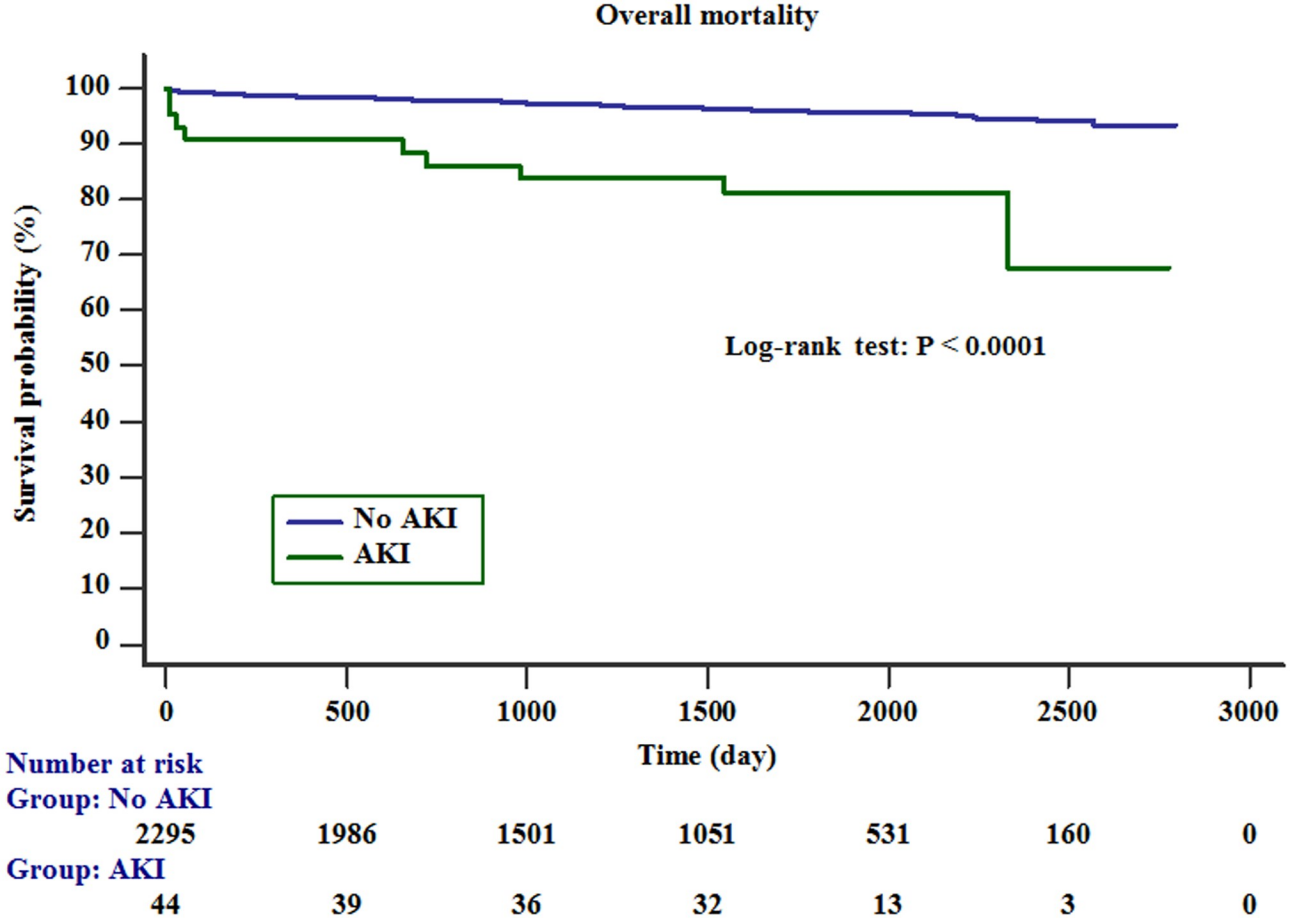
Kaplan–Meier survival curve. The survival rate was significantly lower in patients with AKI than in those without AKI (log-rank test; p < 0.001).

The survival rate of the patients with AKI was significantly lower than that of the patients without AKI (Kaplan–Meier method and log-rank test; p <0.01) (Fig 2).

## Discussion

We investigated whether preoperative albumin levels affected postoperative outcomes, including postoperative AKI, ICU and hospital stay, and overall mortality inpatients who underwent aneurysm clipping surgery. We found that a low preoperative albumin level (≤3.9 g/dL) was associated with postoperative AKI and longer hospital stay, but not with overall mortality. After PS matching analysis, a lower preoperative albumin level was associated with AKI development (p <0.01) and overall mortality (p = 0.04). Multivariate analysis revealed that AKI was an independent predictor of overall mortality (p = 0.03).

Previous studies have reported the risk factors of AKI after hemorrhagic or traumatic brain injury [12, 13]. However, there is little information on the risk factors for postoperative AKI following aneurysm clipping surgery. Our present study revealed that lower preoperative albumin level, male sex, low hemoglobin level, and aneurysmal SAH are associated with AKI following aneurysm clipping surgery. Intraoperative phenylephrine infusion effectively prevented AKI.

Previous studies have reported an association between hypoalbuminemia and increased risk of postoperative AKI [8, 15, 16]. The relationship of hypoalbuminemia to postoperative AKI might be attributable to the renoprotective properties of albumin. Albumin improves renal perfusion and glomerular filtration through prolonged potent renal vasodilation, which is induced by serum albumin reacting with the oxides of nitrogen to form S-nitroso-albumin [17]. Moreover, albumin inhibits apoptosis in renal tubular cells by scavenging reactive oxygen species and carrying protective lysophosphatidic acid [18]. Albumin enhances the proliferation of renal tubular cells through the activation of phosphatidylinositide 3-kinase [19]. In addition, albumin has a ligand-binding capacity and can mitigate the effects of nephrotoxic mediations [20]. Due to these properties, albumin is crucial for maintaining the structural integrity and function of the proximal tubule; thus, a lower level of serum albumin may increase the risk of postoperative AKI. Moreover, recent reports suggest that exogenous albumin administration is beneficial for protecting the kidneys from AKI [21, 22].

Although postoperative AKI develops by a multifactorial mechanism, one of the most common causes is acute tubular necrosis due to hypoxic damage to the nephrons in the medulla of the kidney, which may be induced by hypotension, hypovolemia, and/or dehydration [23]. A decrease in the effective circulating volume leads to the activation of vasoconstriction and salt-retaining neurohumoral systems such as the sympathoadrenal system, and increase in angiotensin, aldosterone, and antidiuretic hormone [23]. The resultant reabsorption of salt and water in the medullary thick ascending limb is associated with an increased demand for oxygen and induces hypoxic injury to the medullary region [24, 25]. Albumin might exert protective effects against acute tubular necrosis, which results from hypoxic injury to the renal medulla. Therefore, administration of exogenous albumin may benefit patients with low preoperative albumin levels. However, exogenous albumin has been reported to be associated with increased mortality in patients with traumatic brain injury [26], which may be due to albumin-induced increases in intracranial pressure [27]. This, along with previous concerns regarding the administration of exogenous albumin, may induce increases in interstitial colloid osmotic pressure and increase intracranial pressure by the extravasation of albumin through the damaged blood-brain barrier [28]. Nevertheless, the status of the blood-brain barrier has not been investigated in patients who undergo aneurysm clipping surgery. Therefore, further prospective studies are needed to identify the clinical effects of exogenous albumin administration to patients with low albumin levels who undergo aneurysm clipping surgery.

In the present study, aneurysmal SAH was also found to be associated with AKI. Dysfunction of non-neurological organs has been correlated with the extent of neurological impairment [29]. Patients with aneurysmal SAH have greater risk of diminished mental capacity, and thus require intubation or surgical or endovascular procedures. As a result, patients with aneurysmal SAH may frequently require radiographic studies and interventions with radiocontrast agents, as well as hyperosmolar therapeutic agents and nephrotoxic antibiotics, which carry greater risks of AKI.

In the present study, an intraoperative phenylephrine infusion was shown to effectively prevent AKI. Although phenylephrine was used more often in hypoalbuminemic patients, it was associated with a low rate of AKI. In fact, there has been concern that vasopressor use is accompanied by renal vasoconstriction and AKI. However, there is also a rationale for vasopressor therapy use in hypotensive states [30]: physiologically, in all regional circulation including renal, splanchnic, cerebral, and coronary beds, blood flow is pressure-dependent outside of the levels of pressure that remain within the autoregulation values for a given regional circulation. This means that if the cardiac output is preserved, organ blood flow is also preserved as long as sufficient blood pressure is maintained. If blood pressure falls below an autoregulatory threshold, organ blood flow also decreases in an almost linear fashion. Especially for the kidneys, the autoregulatory threshold is a mean arterial pressure of 80 mmHg, which is higher than those of the brain or heart, and the risk of ischemic injury is greater. Therefore, it is essential to maintain the mean arterial pressure to preserve renal blood flow. This may be the underlying mechanism of why phenylephrine infusion was helpful for preventing AKI.

Our study had several limitations. First, due to the retrospective design of these analyses, we could not control for all confounding parameters that might have affected our results. Although we performed IPTW analysis to reduce for selection bias, we could not control every residual confounding factor. Second, because we enrolled patients who underwent aneurysm-clipping surgery, our results may not be directly applicable for other types of patients, and data interpretation should thus be performed with care.

In conclusion, a preoperative albumin level of ≤ 3.9 g/dL was associated with postoperative AKI and overall mortality in patients who underwent aneurysm clipping surgery. In addition, postoperative AKI was related to overall mortality following aneurysm clipping surgery.

## Supporting information

S1 Table Patients’ perioperative characteristics and outcomes stratified by subarachnoid hemorrhage status. (DOCX)

S1 Dataset (XLSX)

## Abbreviations

AKI: Acute kidney injury
CCB: Calcium channel blocker
DM: Diabetes mellitus
eGFR: Estimated glomerular filtration rate
HR: Hazard ratio
HTN: Hypertension
ICU: Intensive care unit
IHD: Ischemic heart disease
KDIGO: Kidney disease improving global outcome
PS: propensity score
SAH: subarachnoid hemorrhage
sCr: Serum creatinine
