# Supplementary material for: Impact of the serum albumin level on acute kidney injury after cerebral artery aneurysm clipping
Source: PLoS One. 2018 Nov 5;13(11):e0206731. doi: 10.1371/journal.pone.0206731 (PMC6218058; doi:10.1371/journal.pone.0206731)
Supplement: S1 Table — (DOCX) [file pone.0206731.s001.docx]

Supplementary Table. Perioperative characteristics and outcomes of patients stratified by subarachnoid hemorrhage status

| **Aneurysm** | **Without SAH (N=1982)** | **With SAH (N=357)** | **Total (N=2339)** | **p** |
| --- | --- | --- | --- | --- |
| **Demographics** |  |  |  |  |
| Male, n (%) | 608 (30.7%) | 121 (33.9%) | 729 (31.2%) | 0.25 |
| Age, yr | 56.5 ± 9.6 | 54.8 ± 11.9 | 56.3 ± 10.0 | <0.01 |
| BMI, kg/m^2^ | 24.7 ± 3.2 | 23.4 ± 3.1 | 24.5 ± 3.3 | <0.01 |
| DM, n (%) | 206 (10.4%) | 7 (2.0%) | 213 (9.1%) | <0.01 |
| HTN, n (%) | 985 (49.7%) | 60 (16.8%) | 1045 (44.7%) | <0.01 |
| IHD, n (%) | 89 (4.5%) | 9 (2.5%) | 98 (4.2%) | 0.12 |
| CCB, n (%) | 785(39.6%) | 158 (44.3%) | 943 (40.3%) | 0.11 |
| ACEI, n (%) | 520 (26.2%) | 15 ( 4.2%) | 535 (22.9%) | <0.01 |
| Beta blocker, n (%) | 282 (14.2%) | 24 ( 6.7%) | 306 (13.1%) | <0.01 |
| Aspirin, n (%) | 205 (10.3%) | 29 ( 8.1%) | 234 (10.0%) | 0.23 |
| Anti-platelet, n (%) | 258 (13.0%) | 16 ( 4.5%) | 274 (11.7%) | <0.01 |
| Statin, n (%) | 438 (22.1%) | 10 ( 2.8%) | 448 (19.2%) | <0.01 |
| **Preoperative laboratory findings** | |  |  |  |
| Hemoglobin, g/dL | 13.3 ± 1.4 | 13.3 ± 1.8 | 13.3 ± 1.5 | 0.85 |
| Albumin, g/dL | 4.1 ± 0.3 | 3.9 ± 0.4 | 4.1 ± 0.4 | <0.01 |
| Creatinine., mg/dL | 0.8 ± 0.2 | 0.7 ± 0.2 | 0.7 ± 0.2 | < 0.01 |
| eGFR (ml/min/1.73m^2^) | 736. ± 13.3 | 72.8 ± 14.2 | 73.5 ± 13.4 | 0.34 |
| Uric acid. Mg/dL | 4.7 ± 1.4 | 4.3 ± 1.5 | 4.6 ± 1.4 | <0.01 |
| Na^+^, mmol/L | 141.0 ± 2.3 | 138.3 ± 3.5 | 140.6 ± 2.7 | <0.01 |
| K^+^, mmol/L | 4.1 ± 0.3 | 3.9 ± 0.5 | 4.1 ± 0.4 | <0.01 |
| Cl^-^, mmol/L | 104.2 ± 2.6 | 103.9 ± 3.8 | 104.2 ± 2.8 | 0.18 |
| **Intraoperative variables** | |  |  |  |
| Crystalloid. mL | 1845.7 ± 790.6 | 2258.3 ± 1300.9 | 1908.5 ± 899.4 | <0.01 |
| Mannitol. mL | 77.9 ± 54.4 | 122.8 ± 65.6 | 85.8 ± 59.0 | <0.01 |
| Diuretics, n (%) | 26 (1.3%) | 8 (2.2%) | 34 (1.5%) | 0.26 |
| Urine output, mL | 781.0 ± 518.8 | 882.4 ± 720.4 | 796.4 ± 555.3 | 0.01 |
| Anesthetic time, min | 279.2 ± 73.9 | 323.2 ± 89.5 | 285.9 ± 78.1 | <0.01 |
| Packed RBC, n (%) | 118 (6.0%) | 54 (15.2%) | 172 (7.4%) | <0.01 |
| Lowest SBP, mmHg | 98.3 ± 8.3 | 92.8 ± 9.0 | 93.1 ± 8.4 | 0.59 |
| MBP, mmHg | 63.6 ± 5.5 | 63.8 ± 6.5 | 63.6 ± 5.7 | 0.61 |
| **Outcome variables** | |  |  |  |
| AKI, n (%) | 22 (1.1%) | 22 (6.2%) | 44 (1.9%) | <0.01 |
| ICU admission, n (%) | 1869 (94.3%) | 344 (96.4%) | 2213 (94.6%) | 0.14 |
| ICU stay, day | 1.0 [1.0‒1.0] | 3.0 [2.0‒8.0] | 1.0 [1.0‒1.0] | <0.01 |
| Hospital stay | 6.0 [5.0‒7.0] | 15.0 [10.0‒27.0] | 6.0 [5.0‒9.0] | <0.01 |
| Mortality, n (%) | 52 (2.6%) | 35 (9.8%) | 87 (3.7%) | <0.01 |

SAH, subarachnoid hemorrhage status; BMI, body mass index; DM, diabetes mellitus; HTN, hypertension; IHD, ischemic heart disease, CCB; calcium channel blocker; ACEI, angiotensin-converting enzyme inhibitor; eGFR, estimated glomerular filtration rate; RBC, red blood cells; SBP, systolic blood pressure; MBP, mean blood pressure; AKI, acute kidney injury by KDIGO criteria; ICU, intensive care unit
